# ^75^As Nuclear Magnetic Resonance Spectroscopic Investigation of the Thioarsenate Speciation in Strongly Alkaline Sulfidic Leaching Solutions

**DOI:** 10.3390/molecules29122848

**Published:** 2024-06-14

**Authors:** Erica Brendler, Karsten Meiner, Jörg Wagler, Alexandra Thiere, Alexandros Charitos, Michael Stelter

**Affiliations:** 1 Institute of Analytical Chemistry, TU Bergakademie Freiberg, Lessingstr. 45, 09599 Freiberg, Germany; 2 Institute for Nonferrous Metallurgy and Purest Materials, TU Bergakademie Freiberg, Leipziger Str. 34, 09599 Freiberg, Germany; karsten.meiner1@inemet.tu-freiberg.de (K.M.); alexandra.thiere@inemet.tu-freiberg.de (A.T.); alexandros.charitos@inemet.tu-freiberg.de (A.C.); stelter@inemet.tu-freiberg.de (M.S.); 3 Institute of Inorganic Chemistry, TU Bergakademie Freiberg, Leipziger Str. 29, 09599 Freiberg, Germany; joerg.wagler@chemie.tu-freiberg.de

**Keywords:** tetrathioarsenate(V), ^75^As NMR, oxothioarsenate(V), alkaline leaching, arsenic-rich concentrates, complex copper concentrates

## Abstract

Copper ores and concentrates thereof feature an increasingly notable content of impurities such as arsenic and other hazardous elements. As an alternative to the state-of-the-art partial roasting process, arsenic could be removed by the alkaline sulfide leaching of the copper concentrates. In order to optimize and understand the processes, knowledge of the speciation and oxidation states is essential. In addition to methods such as UV/Vis spectroscopy, chromatography and ICP/MS methods, ^75^As NMR spectroscopy may be useful for the differentiation and quantification of the various species. Although arsenate(V) has been characterized by ^75^As NMR some time ago, to our knowledge, there are no data on tetrathioarsenate(V) AsS_4_^3−^ and the mixed oxygen/sulfur substituted mono-, di- and trithioarsenates(V) AsO*_x_*S_4−*x*_^3−^, *x* = 3, 2, 1, respectively. Therefore, we investigated several model solutions and samples from Cu-As leaching with ^75^As NMR. The strongly alkaline conditions of the leaching solution proved to be very advantageous for that purpose. Both the tetrathioarsenate(V) and the mixed species AsO*_x_*S_4−*x*_^3−^ (*x* = 1–3) could be characterized and provide valuable data for the quantification of the material flows in the leaching process.

## 1. Introduction

Because of their toxicity, arsenic compounds are the subject of numerous studies. They are liberated into ecosystems by geochemical processes, but they are also released by humans through mining and the extraction of metals [1,2]. Another source of arsenic-based environmental pollution are residues of arsenic containing chemical warfare agents [3,4], and various degradation products of inorganic and organic arsenicals, which have been used or are still used in medicine to treat certain diseases like cancer [5,6]. Therefore, arsenic is a common trace element in natural waters, which can also have elevated concentrations in certain geochemical settings [1]. Physiological hazards arising from arsenic compounds are mainly due to their chalcophilicity, resulting in a high affinity of arsenic compounds to react with thiol groups. The speciation of the thioarsenates thus formed is investigated with various methods, with ion chromatography (IC) among them [7], combined with inductively coupled plasma mass spectrometry (IC-ICP/MS) [1], capillary zone electrophoresis [8], or even visible–UV absorption spectra [9] and Raman spectroscopy [10]. A very good overview of the occurrence of thiolated arsenic in natural systems, its reactions and the methods, devices and measurement conditions used to analyze these species can be found in the works of Herath and Leermakers et al. [2,11], and for arsenic species in general, in the works of Radke and Gupta et al. [12,13].

If arsenic is substituted with organic residues, NMR spectroscopy can be used to characterize and identify the compounds by recording and evaluating the ^1^H and ^13^C spectra of these substituents [14,15,16,17]. The direct detection of arsenic in the compounds would be valuable but is challenging. Even though ^75^As is a 100% isotope with a sensitivity of well over two orders of magnitude higher than that of ^13^C [18], its detection is hampered by various effects. Several influences result in a very efficient (in terms of too fast) relaxation leading to a severe line broadening, which can be so strong that the signals can no longer be observed in solution NMR. One reason is the fast exchange between the different dissociation levels within the protolysis equilibria of arsenite and arsenate. Another reason results from the fact that the ^75^As isotope is a quadrupolar nucleus (nuclear spin of I = 3/2), and thus possesses an electric quadrupolar moment Q, which can interact with the electric field gradient (EFG) about the nucleus. As the molecule tumbles in solution, the EFG is modulated and these fluctuations can stimulate relaxation. This contribution is particularly efficient for a large EFG due to different substituents or free electron pairs, resulting in high quadrupolar coupling constants C_Q_. Hence, because of the presence of an As-located lone pair, As(III) compounds can hardly be observed. In contrast, uniformly substituted arsenic(V) compounds in tetrahedral (AsO_4_^3−^) or octahedral coordination (AsF_6_^−^) can be easily observed due to the significantly lower EFG associated with the cubic symmetry of their As coordination spheres. Thus, ^75^As NMR data of such compounds are described in the literature, with the latter even being used as a chemical shift standard for arsenic [18].

Nevertheless, ^75^As NMR investigations in solution are rare. Reviews are provided by Klapötke and Pettinari et al. [19,20] and range from the investigation of arsenic salts in general [21], As(OTeF_5_)_5_ and As(OTeF_5_)_6_^−^ in solution [22], Keggin ions [23], and arsenic oxy salts [24], to the AsO_4_^3−^ interaction with biomolecules [25]. Surprisingly, ^75^As chemical shift data could not be found for thioarsenates in solution, not even for the tetrathioarsenate [20]. One reason could be that arsenate forms poorly soluble As_2_S_5_ under neutral and acidic conditions in the presence of sulfide ions. These can only be dissolved by sulfide ions under alkaline conditions.

The same also applies to solid-state NMR. Although copper tetrathioarsenate (Enargite, Cu_3_AsS_4_) has been investigated by solid state ^75^As NMR and NQR [26], surprisingly, no chemical shift data were provided in that reference. Investigations of antiferromagnetic and superconducting materials [27], semiconductors [28], and quantum computer materials [29] are topics of continuous interest. To date, arsenic oxysalts have already been well characterized in solid state [24], which was possible because of enhanced magnetic field strengths B_0_, which became available during the last two decades. Investigations at high magnetic fields are advantageous in solution and solid state because the second order quadrupolar contributions, which are mainly responsible for the fast relaxation and broad lines, are reduced with increasing B_0_ [30]. Quadrupolar constants of, e.g., C_Q_ = 2.85 MHz compared to 5.1 MHz, measured for an alkaliarsenate or an alkalidihydrogenarsenate, respectively, show the influence of charge symmetry on the quadrupole interactions described above [24].

As an effect of the chalcophilicity of arsenic, it usually occurs in sulfide ores, and therefore, also as an accompanying element in copper ores. With the depletion of copper-rich deposits, the content of arsenic and other environmentally harmful elements in ores and their concentrates is increasing; hence, the challenge of separating them also increases. As an alternative to the state-of-the-art partial roasting process to deplete these secondary components, arsenic could be removed by the alkaline sulfide leaching of the copper concentrates [31,32,33,34,35,36]. In order to optimize and understand the processes, knowledge of the speciation and oxidation stages is essential.

^75^As NMR studies of H_3_AsO_4_/Na_3_AsO_4_ solutions depending on pH showed that, at pH values above 13.5, the ^75^As NMR line became much narrower due to a shift in the dissociation equilibrium to the completely deprotonated, symmetrical tetrahedral anion [25]. Because the separation of arsenic in copper production is performed in strongly alkaline sulfidic solutions, in which the species should also be dominated by completely deprotonated arsenate derivatives, the use of ^75^As NMR in combination with high magnetic fields appears to be promising. Therefore, we investigated leaching samples and arsenic(V) sulfide model solutions at different compositions and pH values to evaluate the suitability of the method for the qualitative and quantitative description of arsenic speciation in these solutions and to determine the chemical shifts of the thioarsenates(V) AsO*_x_*S_4−*x*_^3−^ (*x* = 0–3) in solution. 

## 2. Results and Discussion

**Figure 1:**
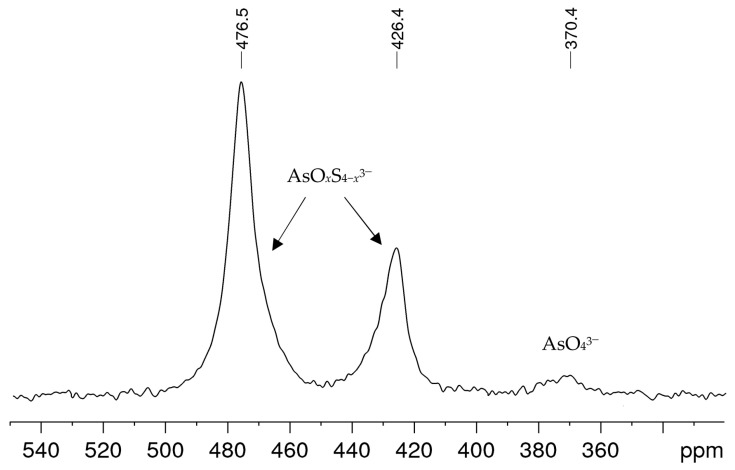
^75^As NMR spectrum of an alkaline leaching solution obtained from an ore concentrate treated for 4 h with an alkaline sulfide leach (ASL) prepared from 2.5 M NaOH and 1.5 M Na_2_S·3H_2_O. The solution contained 3.18 g/L arsenic.

In alkaline sulfide leaching, ores or ore concentrates are treated with solutions obtained from 1 M–3 M caustic soda NaOH and sodium sulfide Na_2_S·3H_2_O, sometimes with the addition of elemental sulfur, in order to separate arsenic from the mixture of sulfide ores. Figure 1 shows a typical ^75^As NMR spectrum obtained from a leaching solution with an arsenic concentration in the medium range within the solutions examined. Besides a small signal at *δ =* 370 ppm, which can be attributed to arsenate(V) AsO_4_^3−^ [21], two further signals were observed at 426 and 476 ppm. These signals could not be interpreted from the literature data, even though the solution composition and history suggest that they could be arsenic sulfide species. All alkaline leaching solutions investigated showed the same signals, only the proportions and line widths varied. In spectra with comparatively smaller linewidths, as in the example shown here, an asymmetry was often observed for the two unknown signals, which indicates a superposition of signals.

Model solutions were therefore produced and examined in order to enable and verify the assignment of the signals. The measurements of model solutions started with a solution of the tetrathioarsenate anion AsS_4_^3−^ prepared by dissolving freshly precipitated As_2_S_3_ in an aqueous ammonium polysulfide (NH_4_)_2_S*_x_* solution [37,38].

As_2_S_3_ + 6 SH^−^ + 2 S ⇌ 2 AsS_4_^3−^ + 3 H_2_S (1)

**Figure 2:**
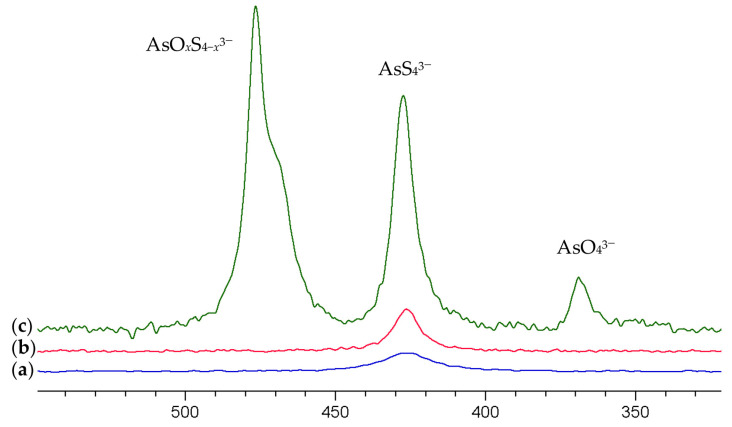
^75^As NMR spectra of (**a**) a 0.25 M (NH_4_)_2_AsS_4_ solution (**b**) diluted by 20% with 1 M NaOH (**c**) 10-fold dilution with 1 M NaOH. The spectra are scaled to equal the concentration of the solution.

Figure 2a shows the resulting ^75^As NMR spectrum of the solution which exhibits a signal at 427 ppm. As the preparation of the arsenate solution involved sulfide species only, the signal can clearly be assigned to AsS_4_^3−^. 

In order to produce oxothioarsenates, the mother solution was gradually diluted by the addition of a sodium hydroxide (NaOH) solution. The ion chromatograms reported by Stauder et al. [1,39], who tried to synthesize stock solutions of the individual oxothioarsenates, already showed that a single species does not exist in an alkaline solution; instead, a distribution of oxothioarsenates can be found depending on the sulfide-to-hydroxide ratio. AsS_4_^3−^ can be converted to oxothioarsenates by the following reactions [40,41,42]:

AsS_4_^3−^ + 2 OH^−^ ⇌ AsOS_3_^3−^ + S^2−^ + H_2_O (2)

AsOS_3_^3−^ + 2 OH^−^ ⇌ AsO_2_S_2_^3−^ + S^2−^ + H_2_O (3)

After two further analogous steps, they provide the following overall reaction:

AsS_4_^3−^ + 8 OH^−^ ⇌ AsO_4_^3−^ + 4 S^2−^ + 4 H_2_O. (4)

The resulting spectra are shown in Figure 2b,c. Increasing the pH by a dilution of 20% with 1 M NaOH (pH approx. 13) provides a slightly more intense (+35%) and narrower signal for AsS_4_^3−^. Increasing the pH further results in an extreme increase in the signal intensity by a factor of 16.7 (+1570%!) and the occurrence of a further signal at 476.6 ppm with a shoulder at about 470 ppm, which we attribute to oxothiorsenate anions AsO_4−*x*_S*_x_*^3−^ (*x* = 1–3) and the signal of arsenate AsO_4_^3−^ at 369 ppm. 

Obviously, only a small fraction of the arsenic speciation can be observed in the spectra shown in Figure 2a,b. This can be rationalized by the dissociation equilibria for these compounds. Dissociation constants have been reported by Brookins et al. and Thilo et al. [43,44] and are summarized in the Supplementary Materials in Table S1. The calculation of the species distribution of the dissociation stages (see Supplementary Materials, Figure S1 for the compounds where the pK values of all dissociation stages are known) shows clearly that even at a pH of 13–14, a significant share of the arsenate and oxothioarsenates is still protonated. Only the tetrathioarsenate has a much smaller dissociation constant, resulting in a large share of completely deprotonated anions at less alkaline pH values. The larger electric field gradient at protonated arsenate species and the fast proton exchange between the species of the different dissociation stages results in a severe line broadening due to exchange-driven faster relaxation and due to quadrupolar interaction, making a great share of the arsenic invisible for NMR if the pH is not high enough. This is an effect well known also for other quadrupolar nuclei, e.g., the cations of group 13 metals aluminum, indium and gallium [45,46]. It has also been described for arsenate AsO_4_^3−^ solutions studied at variable pH values by Geraldes et al. [25] who observed a noticeable sharpening of the ^75^As NMR line at a pH > 13.5. 

**Figure 3:**
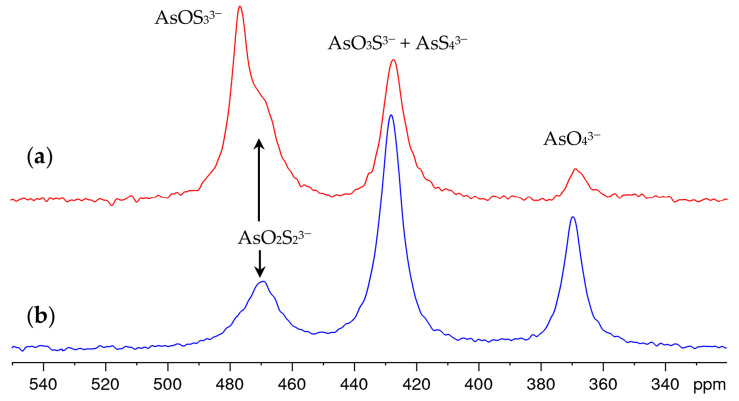
^75^As NMR spectra of 0.25 M (NH_4_)_2_AsS_4_ solution after 10-fold dilution with 1 M NaOH (**a**) freshly prepared solution (**b**) aged solution (upon storage under air at atmospheric pressure and 20–25 °C for 11 months).

Time-dependent measurements showed that the sulfide–hydroxide exchange in the solution equilibrated within one hour after the addition of NaOH and that the composition did not change significantly within one day. Over a period of weeks and months, however, oxidation causes the tetrathioarsenate and the oxothioarsenates to degrade in favor of the arsenate. As a representative example, Figure 3 shows the spectrum of the solution discussed in Figure 2c, freshly prepared and after aging. The oxothioarsenate signal at 479 ppm is the strongest signal in this solution but also in other investigated mixtures with a high share of tetrathioarsenate, and is reduced at a higher arsenate share. Therefore, we conclude that at 479 ppm, the oxotrithioarsenate AsOS_3_^3−^ can be observed. It is no longer visible in the aged solution. The remaining signal at 470 ppm corresponds to the previously observed shoulder and is therefore assigned to dioxodithioarsenate AsO_2_S_2_^3−^.

The arsenate content (signal at 369 ppm) increases, as does the signal at 427 ppm, which was originally assigned to the tetrathioarsenate only. As expected, the overall intensity of the spectra remains constant. Since it is chemically unlikely that the concentration of tetrathioarsenate increases again under these strongly alkaline conditions, this suggests that the signals of tetrathioarsenate and trioxothioarsenate overlap at 427 ppm. This is also supported by the fact that the signal maximum shifts from 427 ppm by approx. 1 ppm to higher values during aging, in contrast to the other signals.

In some of the aged solutions, in addition to the increased oxothioarsenate and arsenate share, an increase in line width was also observed. The broader lines can be explained by the hydroxide consumption within the underlying reactions (see Equations (2)–(4)) reducing the pH of the solutions, and thus, the share of deprotonated oxothioarsenate species.

According to Herath et al. [2], the sulfide can also be oxidized, additionally reducing the share of thioarsenates, and again, the pH in solution, e.g., as follows:

AsO_2_S_2_^3−^ + 2 O_2_ + 2 OH^−^ ⇌ AsO_3_S^3−^ + 2 SO_4_^2−^ + H_2_O (5)

The signals of AsOS_3_^3−^ and AsO_2_S_2_^3−^ overlap and result in a broad, not resolved, asymmetric resonance at 476 ppm. This can be observed similarly for the signals of AsS_4_^3−^ and AsO_3_S^3−^, whereby these overlap even more significantly due to similar chemical shifts.

The line width of the resonances of quadrupolar nuclei in solution can be reduced by increasing the measuring temperature. The thus induced faster molecular tumbling influences the fluctuation of the EFG and can make this relaxation contribution become less efficient, resulting in longer relaxation times, narrower signals and better resolution of the signals in the spectra [47,48]. Since the resonances of quadrupolar nuclei are very broad, these changes are evident, in contrast to the situation for other relaxation mechanisms. This effect was also observed for solutions of arsenic salts [21]. 

Solutions with a high proportion of these mixed oxothioarsenate species were therefore selected to be examined at elevated temperatures. Note that the measurement of highly alkaline solutions in glass tubes, especially at elevated temperatures, is risky due to the alkaline attack at the glass. This effect can be reduced by working with quartz glass tubes or even be prevented by using Teflon liners. 

Since the sulfide content in polysulfide solutions is difficult to control, these specific mixtures of different oxothioarsenates were prepared by dissolving arsenic(V) sulfide As_2_S_5_ in the sodium hydroxide solution [37]. Equation (6) provides the first step in the dissolution reaction; the oxothioarsenates can equilibrate and further react according to Equations (3) and (4).

As_2_S_5_ + 6 OH^−^ ⇌ AsOS_3_^3−^ + AsO_2_S_2_^3−^ + 3 H_2_O (6)

**Figure 4:**
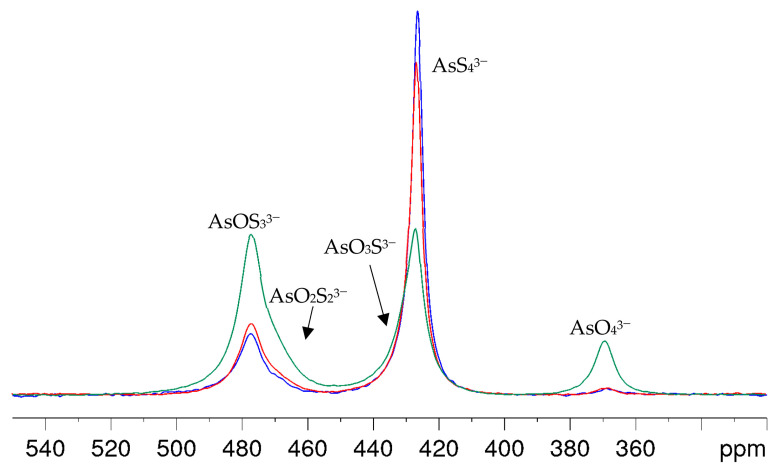
^75^As NMR spectra of a solution with As/OH = 1:8 molar ratio (0.125 M As in 1 M NaOH) within 1 h (blue), after 3 h (red) and 6 d (green) after preparation.

With arsenic-to-hydroxide ratios close to the stoichiometric conversion in Equation (6), the formation of the oxothioarsenates took a longer time than was appropriate for the intended NMR investigations to achieve an even distribution of the aimed species. Even several hours after the preparation of the A_2_S_5_-NaOH mixture, the spectra showed mainly AsS_4_^3−^ and small amounts of AsOS_3_^3−^ and AsO_2_S_2_^3−^_._ In addition, the NMR signals became broad due to the consumption of the hydroxide and the resulting lower pH value of the solutions. Even a solution corresponding to an OH/As molar ratio of eight showed only a slow conversion, as can be clearly seen in Figure 4. Within one hour after synthesis, hardly any arsenate and only a small amount of oxothioarsenates can be observed. After 3 h, the percentages have increased only slightly, but a clear change in favor of the oxothioarsenates and AsO_4_^3−^ can only be seen after six days. For better visibility of the changes in line shapes and positions, a stacked plot of the spectra in Figure 4 is provided in the Supplementary Materials with Figure S2. The intensity of AsOS_3_^3−^ and AsO_2_S_2_^3−^ increases, the amplitude of the AsS_4_^3−^ signal decreases, but the signal becomes broader and asymmetric due to the formation of AsO_3_S^3−^. Again, this overlapping signal slightly shifts to a higher chemical shift.

A suitable composition of the solutions for the measurements at elevated temperatures resulted from the following consideration. If all the sulfur was converted to tetrathioarsenate, the balance would be as follows:

4 As_2_S_5_ + 24 OH^−^ ⇌ 5 AsS_4_^3−^ + 3 AsO_4_^3−^ + 12 H_2_O (7)

In order to theoretically obtain a mixture with 50% sulfur and 50% oxygen on arsenic, two equivalents of arsenic in the form of sodium arsenate(V) solution were added to the mixture. The concentration of arsenic was set to 0.3 M in order to reduce the time necessary to record the spectra at an elevated temperature. 

The AsO*_x_*S_4−_*_x_*^3−^ (*x* = 0–4) species distribution shows a sufficiently stable composition 10 h after preparation, as can be seen in the Supplementary Materials in Figure S3. 

**Figure 5:**
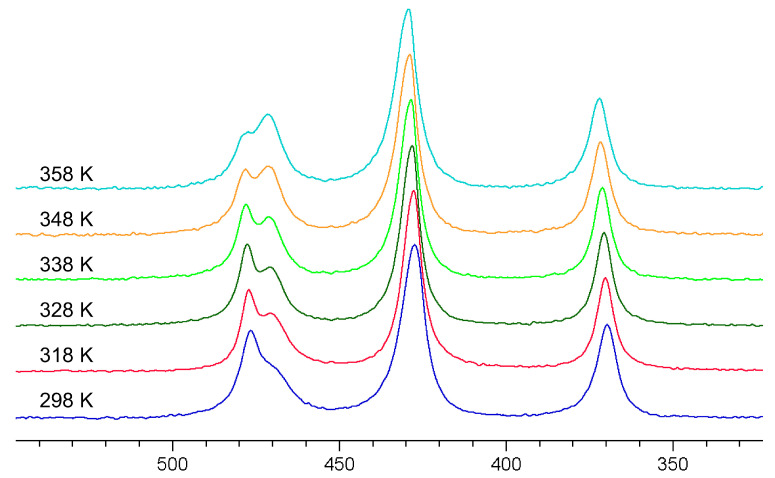
^75^As NMR spectra of a 0.3 M As solution (0.12 mol As_2_S_5_ + 0.06 mol Na_3_AsO_4_ in 2 M NaOH) at 298, 318, 328, 338, 348, and 358 K (bottom to top).

Figure 5 shows the spectra of the AsO*_x_*S_4−_*_x_*^3−^ (*x* = 0–4) mixture at different temperatures. As can be seen very clearly, the signals of oxotrithioarsenate and dioxodithioarsenate around 470 ppm become significantly narrower, and thus, better-resolved in the temperature range up to 328 K. However, if the temperature is increased further, the conversion to the more oxygen-containing oxothioarsenates is significantly accelerated (see Equation (3)), which can be seen, in particular, in the decrease in oxotrithioarsenate at 476 ppm with the simultaneous increase in dioxodithioarsenate at 471 ppm.

An additional observation was that all signals shifted slightly by nearly +2 ppm due to the temperature dependence of the chemical shift. Unfortunately, the resolution did not improve to the same extent for the overlapping signals of the AsS_4_^3−^ and AsO_3_S^3−^, probably due to the very similar chemical shifts of the two species. The signal only became more clearly asymmetric, as can be seen in the spectrum section in Figure S4 in the Supplementary Materials.

**Figure 6:**
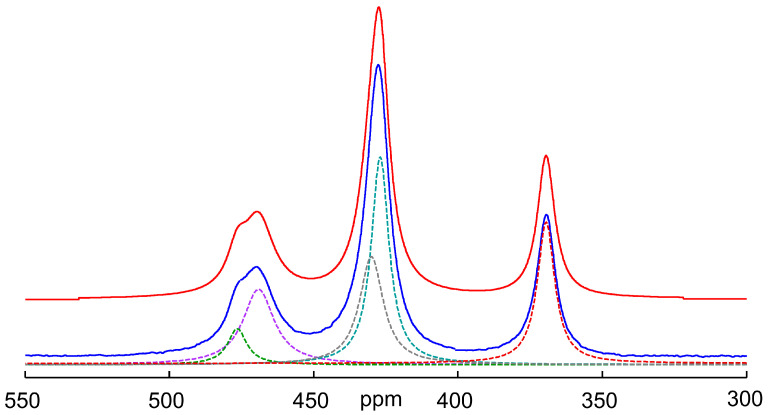
^75^As NMR spectrum of a 0.3 M As solution (0.12 mol As_2_S_5_ + 0.06 mol Na_3_AsO_4_ in 2 M NaOH) at 298 K after temperature-dependent measurements up to 358 K and cooling down again. Blue: experimental spectrum, red: sum of individual peaks, dotted lines: individual signals obtained by deconvolution (for assignment see Figure 4 and cf. Table 1). Number of scans: 4096. The resulting shares in Mol% As are as follows: AsO_4_^3−^/AsO_3_S^3−^ /AsO_2_S_2_^3−^/AsOS_3_^3−^/AsS_4_^3−^ = 21/22/20/6/31.

Figure 6 shows the deconvolution of the spectrum obtained after cooling the solution to 298 K. The chemical shifts and assignment obtained according to this deconvolution attempt are shown in Table 1. As can be seen from the spectra and the table, the chemical shift of the oxothioarsenates AsO*_x_*S_4−*x*_^3−^ (*x* = 0–4) does not change continuously with the exchange of sulfur for oxygen, but the values initially increase and then become smaller and—for the arsenate—even lie below the value of the tetrathioarsenate. This behavior, meaning that the substituent contributions are not constant and can even change signs, has also been observed with other nuclei such as silicon and, for example, with several phosphoric acid derivatives [49,50].

Based on this assignment, the spectra of the alkaline leaching solutions, as shown in the example in Figure 1, can now be interpreted as a mixture of arsenate(V), tetrathioarsenate(V) and the oxothioarsenates(V).

## 3. Materials and Methods

Na_3_AsO_4_ solution: 3.12 g (0.01 Mol) of Na_2_HAsO_4_·7H_2_O (Sigma-Aldrich, Merck KGaA, Darmstadt, Germany) were dissolved in 10 mL of 3.5 M NaOH solution (prepared from NaOH (Fisher scientific, Waltham, MA, USA) and distilled water) to provide a solution 1 M in AsO_4_^3−^. 

Solutions with different OH/As ratios: 37.8/29.1/19.38 mg As_2_S_5_ (0.25/0.185/0.125 mMol As) (As_2_S_5_: stock from preparative practical course, the synthesis was carried out according to [51]) were dissolved in 1 mL of 1 M NaOH (prepared from NaOH (Fisher scientific) and distilled water) resulting in OH to As ratios of 4, 6, 8, respectively.

Solution for variable temperature measurements: 32,4 mg of As_2_S_5_ (0.24 mMol As) and 60 μL of 1 M Na_3_AsO_4_ solution (0.06 mMol As) were dissolved in 940 μL of 2 M NaOH to provide, according to Equation (7), a formal AsS_4_^3−^/AsO_4_^3−^ ratio equal to 1.

Preparation of AsS_4_^3−^ solution: 100 mg (0.505 mmol) of As_2_O_3_ were dissolved in a sufficient volume of 2 M HCl, and As_2_S_3_ was precipitated by the continuous bubbling of H_2_S through this solution for quantitative precipitation. The precipitate was separated from the mother solution by centrifugation, washed with deionized water and dissolved in 4 mL of ammonium polysulfide (NH_4_)_2_S*_x_* solution, providing an approx. 0.25 mM solution of AsS_4_^3−^ with pH = 9. The preparation of the (NH_4_)_2_S*_x_* solution is provided in detail in [38]. 

The leaching sample was prepared with a Chilean copper concentrate (23.1 wt.% Cu; 2.8 wt.% As; 18.8 wt.% Fe; 0.2 wt.% Sb, 0.7 wt.% Pb; 5.3 wt.% Zn) using a leaching solution of 2.5 M NaOH and 1.5 M Na_2_S·3H_2_O at 80 °C with a solid/liquid ratio of 1/5 (40 g concentrate/200 mL solution) and a stirring rate of 250 rpm for 4 h. 

All the experiments of the series, including the leaching example, were carried out in a continuously heated double glass vessel, coupled to an external thermostat and using an overhead stirring motor with a Teflon stirrer and a plastic lid. The sodium hydroxide concentration varied within the series from 1.5 M to 3.5 M NaOH and the sodium sulfide concentration from 0 to 2.0 M Na_2_S·3H_2_O.

The empirically validated standard concentration of the leaching solution was determined to be 2.5 M NaOH and 2.0 M Na_2_S to obtain a residual arsenic concentration of maximum 0.3 wt.%. This threshold applies to every feed concentration at 80 °C, making it competitive with regard to the residual arsenic concentration delivered by the state-of-the-art technology of partial roasting.

NMR spectra were recorded with a Bruker Avance NEO 700 MHz spectrometer, equipped with a 5 mm BBO (broadband observe) probe working at 119.91 MHz for ^75^As. Referencing was performed externally using a 0.5 M solution of KAsF_6_ in acetonitrile-d3 (δ(^75^As) = 0 ppm). The experiment repetition time was 0.5 or 1 s; 2 k to 16 k scans were accumulated to record the spectra. Pulse width of 30° or 60° was applied. Measurements were either performed in coaxial tubes, with the 5 mm tube filled with the sample (500 μL) and the inner tube filled with D_2_O as lock. For variable temperature-dependent measurements quartz glass tubes were used. The use of Teflon liners for 5 mm tubes was also tested. In this case, either a small amount of D_2_O could be added as a lock or the measurements could be performed without a lock, which led to comparable results due to the high line width of the observed signals. Quartz glass tubes or Teflon liners are recommended if working with strong alkaline solutions regularly. The deconvolution of spectra was performed using the program DMFIT (release 20200306) by Massiot et al. [52].

Safety measures: Caution! Sulfide-containing arsenic solutions are toxic. Caustic soda is highly corrosive. All work must be carried out under the fume hood with laboratory gloves. All arsenic-containing solutions were collected and disposed of as heavy metal waste.

## 4. Conclusions

On the basis of the presented investigations, it was possible to characterize tetrathioarsenate and the different oxothioarsenate anions with ^75^As NMR spectroscopy in solution and to determine their ^75^As chemical shifts. Thus, it is now possible to derive the arsenic(V) speciation in alkaline sulfidic leaching solutions (ALS) produced during arsenic separation from ore concentrates, and to monitor the influence of the leaching conditions and the composition of the ALS on the leaching result.

The advantage of using ^75^As NMR spectroscopy as a method for characterizing alkaline leaching solutions is that the solutions can be examined as received. No dilution or pH adjustment, which would interfere with the dynamic equilibrium between arsenite and arsenate species and their different oxygen-sulfur ratios, is necessary to analyze the samples. Together with other methods for determining the total arsenic content using, for example, elemental analytical methods, this opens the possibility of carrying out a balance of the oxidation states and sulfide bound to arsenic under the conditions of alkaline sulfide leaching. Measurements at slightly elevated temperatures are recommended to improve the resolution and evaluability of the spectra, and can be carried out safely and routinely by selecting suitable sample tubes.

## Figures and Tables

**Table 1:** Chemical shifts of the different AsO*_x_*S_4−*x*_^3−^ (*x* = 0–4) species at 298 K.

Arsenate Anion	*δ* (^75^As) in ppm
AsO_4_^3−^	369 ^1^
AsO_3_S^3−^	430
AsO_2_S_2_^3−^	469
AsOS_3_^3−^	476
AsS_4_^3−^	427

^1^ in accordance with [32].

## Data Availability

The original contributions presented in the study are included in the article/Supplementary Material, further inquiries can be directed to the corresponding authors.

## Supplementary Materials

The following supporting information can be downloaded at: https://www.mdpi.com/article/10.3390/molecules29122848/s1, Table S1: Dissociation constants for arsenate, tetrathioarsenate and oxothioarsenates; Figure S1: Species distribution diagrams for the dissociation equilibria; Figure S2: ^75^As NMR spectra of a solution with As: OH = 1:8 molar ratio (0.125 M As in 1 M NaOH) within 1 h, after 3 h and 6 d after preparation; Figure S3: ^75^As NMR spectra of a 0.3 M As solution (0.12 mol As_2_S_5_ + 0.06 mol Na_3_AsO_4_ in 2M NaOH) within 1 h, and after 5, 10, 15, 20 h after preparation; Figure S4: Section of the ^75^As NMR spectrum of a 0.3 M As solution (0.12 mol As_2_S_5_ + 0.06 mol Na_3_AsO_4_ in 2M NaOH) at 358 K. Reference [53] was cited in Supplementary Materials.
