# Supplementary material for: 75As Nuclear Magnetic Resonance Spectroscopic Investigation of the Thioarsenate Speciation in Strongly Alkaline Sulfidic Leaching Solutions
Source: Molecules. 2024 Jun 14;29(12):2848. doi: 10.3390/molecules29122848 (PMC11207089; doi:10.3390/molecules29122848)
Supplement: Supplementary file 1 [file molecules-29-02848-s001.zip › molecules-3015106-supplementary.pdf]

Supporting Information

75As Nuclear Magnetic Resonance Spectroscopic Investigation of the Thioarsenate Speciation in Strongly Alkaline Sulfidic Leaching Solutions

Erica Brendler [1,*], Karsten Meiner [2], Jörg Wagler[3], Alexandra Thiere[2], Alexandros Charitos[2] and Michael Stelter[2]

1 Institute of Analytical Chemistry, TU Bergakademie Freiberg, Lessingstr. 45, 09599 Freiberg, Germany
2 Institute for Nonferrous Metallurgy and Purest Materials, TU Bergakademie Freiberg, Leipziger Str. 34, 09599 Freiberg, Germany; karsten.meiner1@inemet.tu-freiberg.de (K.M.); alexandra.thiere@inemet.tu-freiberg.de (A.T.); alexandros.charitos@inemet.tu-freiberg.de (A.C.); stelter@inemet.tu-freiberg.de (M.S.)
3 Institute of Inorganic Chemistry, TU Bergakademie Freiberg, Leipziger Str. 29, 09599 Freiberg, Germany; joerg.wagler@chemie.tu-freiberg.de
* Correspondence: erica.brendler@chemie.tu-freiberg.de

**Figure/Table**	**Content**	**Page**
Table S1	Dissociation constants for arsenate, tetrathioarsenate and oxothioarsenates	2
Figure S1	Species distribution diagrams for the dissociation equilibria	3
Figure S2	^{75}As NMR spectra of a solution with As : OH = 1 : 8 molar ratio (0.125 M As in 1 M NaOH) within 1 h, after 3 h and 6 d after preparation	4
Figure S3	^{75}As NMR spectra of a 0.3 M As solution (0.12 mol As_2S_5 + 0.06 mol Na_3AsO_4 in 2 M NaOH) within **1** h, and after **5**, **10**, **15**, **20** h after preparation	5
Figure S4	Section of the ^{75}As NMR spectrum of a 0.3 M As solution (0.12 mol As_2S_5 + 0.06 mol Na_3AsO_4 in 2 M NaOH) at 358 K	5
References		6

Table S1. Dissociation constants for arsenate, tetrathioarsenate and oxothioarsenates

Compound	pK_1	pK_2	pK_3	Reference
H_3AsO_4	2.25	6.94	11.59	[43]
H_3AsO_3S	3.3	7.2	11	[44]
$H_3AsO_2S_2$	2.44	7.1	10.9	[44]
H_3AsOS_3	/	/	10.8	[44]
H_3AsS_4	/	/	5.2	[44]

Figure S1. Species distribution diagrams for the dissociation equilibria of a) H_3AsO_4, b) H_3AsO_3S, c) $H_3AsO_2S_2$ as a function of pH with the dissociation levels $H_2AsX_4^{-}$, $HAsX_4^{2-}$, AsX_4^{3-} (X= O, S). H_3AsX_4 was not plotted for clarity as the concentration in the pH range under consideration was essentially zero. The solutions were assumed to be 1 M in arsenic. The calculations were performed with PHREEQC [53]

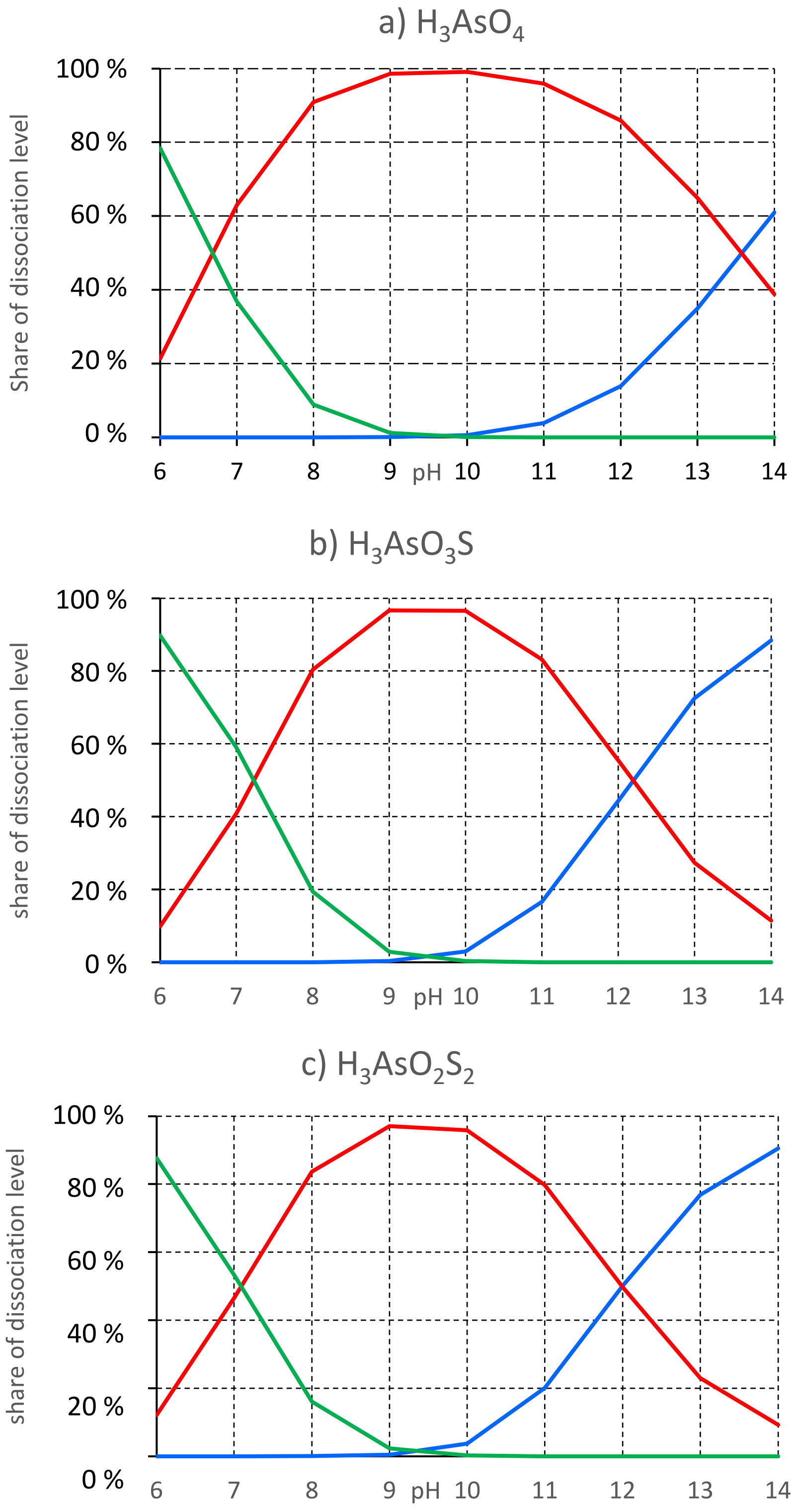

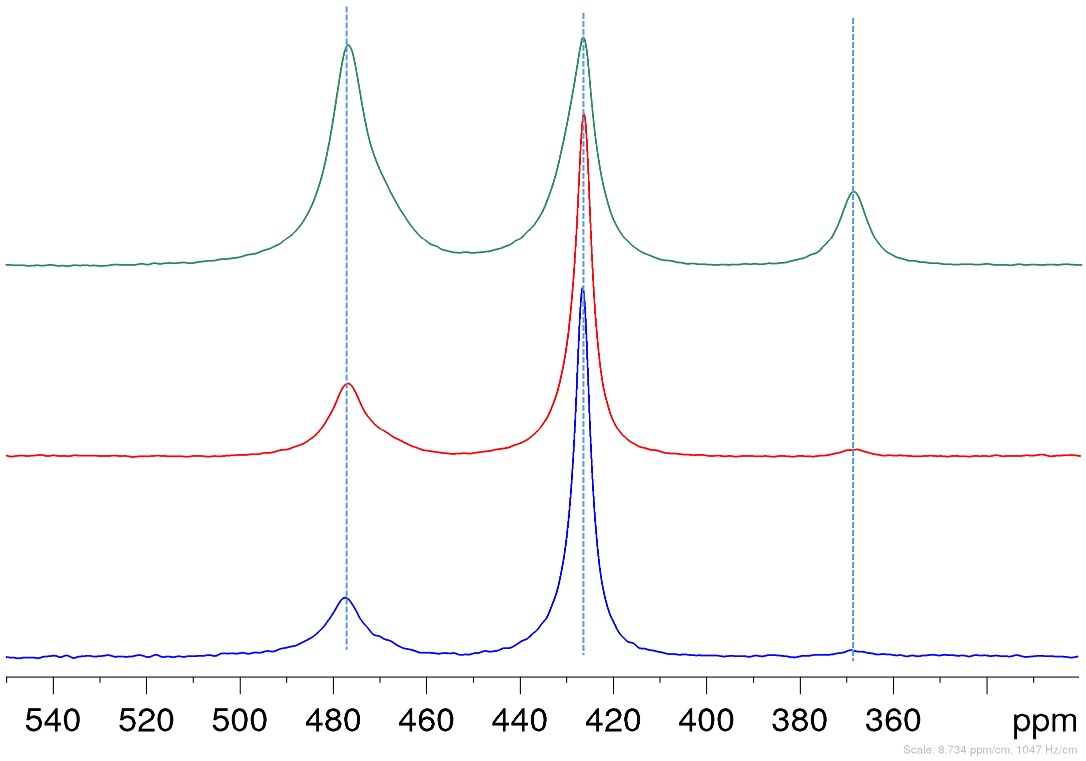

Figure S2. Stack plot of the ^{75}As NMR spectra of a solution with As : OH = molar 1 : 8 ratio (0.125 M As in 1 M NaOH) within 1 h, after 3 h and 6 d after preparation.

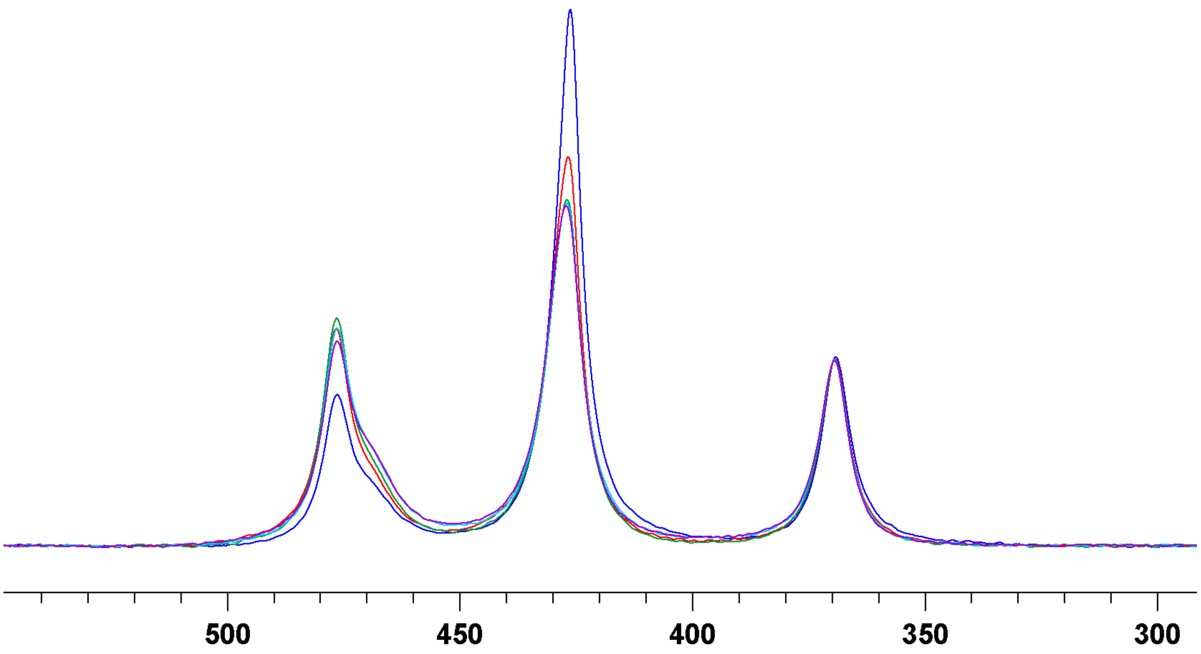

Figure S3. ^{75}As NMR spectra of a 0.3 M As solution (0.12 mol As_2S_5 + 0.06 mol Na_3AsO_4 in 2M NaOH) within **1** h, and after **5**, **10**, **15**, **20** h after preparation. For better comparability, the spectra were scaled to the same intensity for the arsenate signal at 370 ppm.

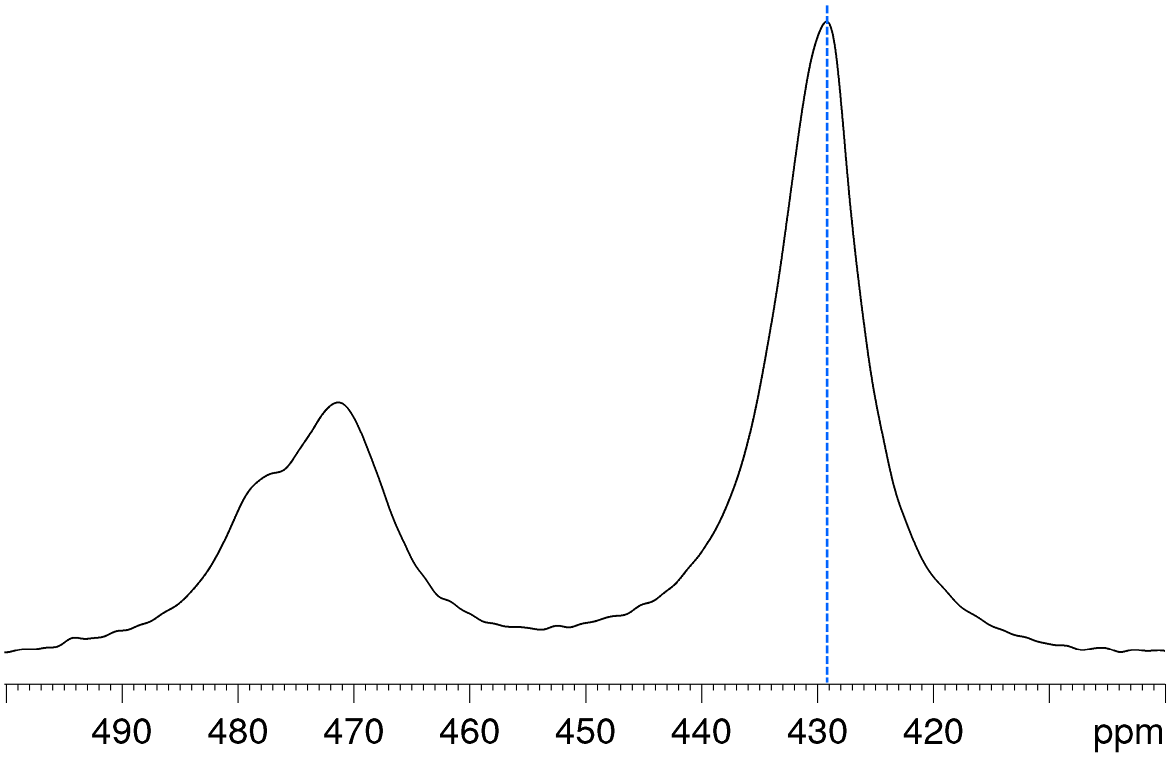

Figure S4: Section of the ^{75}As NMR spectrum of a 0.3 M As solution (0.12 mol As_2S_5 + 0.06 mol Na_3AsO_4 in 2M NaOH) at 358 K (NS = 8192). Note the pronounced asymmetry of the signal at about 429 ppm.

References

43. Brookins, D.G. Arsenic. In *Eh-pH Diagrams for Geochemistry*; Springer: Berlin/Heidelberg, Germany, 1988; pp. 28–29. https://doi.org/10.1007/978-3-642-73093-1_9.

44. Thilo, E.; Hertzog, K.; Winkler, A. Über Vorgänge bei der Bildung des Arsen(V)-sulfids beim Ansäuern von Tetrathioarsenatlösungen. *Z. Anorg. Allg. Chem.* **1970**, *373*, 111–121. https://doi.org/10.1002/zaac.19703730203.

53. Parkhurst, D.L., Appelo, C.A.J. Description of input and examples for PHREEQC version 3—A computer program for speciation, batch-reaction, one-dimensional transport, and inverse geochemical calculations. In *U.S. Geological Survey Techniques and Methods*; Book 6; United States Geological Survey: Reston, VA, USA, 2013; Chapter A43, p. 497.
